# Genomic Selection in the *Era* of Next Generation Sequencing for Complex Traits in Plant Breeding

**DOI:** 10.3389/fgene.2016.00221

**Published:** 2016-12-27

**Authors:** Javaid A. Bhat, Sajad Ali, Romesh K. Salgotra, Zahoor A. Mir, Sutapa Dutta, Vasudha Jadon, Anshika Tyagi, Muntazir Mushtaq, Neelu Jain, Pradeep K. Singh, Gyanendra P. Singh, K. V. Prabhu

**Affiliations:** ^1^Division of Genetics, Indian Agricultural Research InstituteNew Delhi, India; ^2^National Research Centre for Plant BiotechnologyNew Delhi, India; ^3^School of Biotechnology, Sher-e-Kashmir University of Agricultural Sciences and Technology of JammuChatha, India

**Keywords:** genomic selection, GBS, complex traits, GEBVs, crop improvement

## Abstract

Genomic selection (GS) is a promising approach exploiting molecular genetic markers to design novel breeding programs and to develop new markers-based models for genetic evaluation. In plant breeding, it provides opportunities to increase genetic gain of complex traits per unit time and cost. The cost-benefit balance was an important consideration for GS to work in crop plants. Availability of genome-wide high-throughput, cost-effective and flexible markers, having low ascertainment bias, suitable for large population size as well for both model and non-model crop species with or without the reference genome sequence was the most important factor for its successful and effective implementation in crop species. These factors were the major limitations to earlier marker systems viz., SSR and array-based, and was unimaginable before the availability of next-generation sequencing (NGS) technologies which have provided novel SNP genotyping platforms especially the genotyping by sequencing. These marker technologies have changed the entire scenario of marker applications and made the use of GS a routine work for crop improvement in both model and non-model crop species. The NGS-based genotyping have increased genomic-estimated breeding value prediction accuracies over other established marker platform in cereals and other crop species, and made the dream of GS true in crop breeding. But to harness the true benefits from GS, these marker technologies will be combined with high-throughput phenotyping for achieving the valuable genetic gain from complex traits. Moreover, the continuous decline in sequencing cost will make the WGS feasible and cost effective for GS in near future. Till that time matures the targeted sequencing seems to be more cost-effective option for large scale marker discovery and GS, particularly in case of large and un-decoded genomes.

## Introduction

**FIGURE 1:**
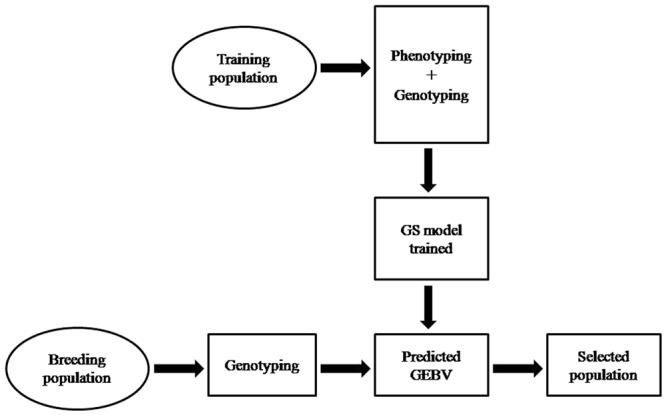
**Showing the different steps of genomic selection (GS) used for crop improvement program**.

Plant breeding has been and will continue remain the major driving force for science based productivity enhancements in major food, feed, and industrial crops. The conventional and marker-assisted breeding (MAB) are the two approaches used to accomplish plant breeding (Breseghello and Coelho, 2013). The conventional breeding involves hybridization between diverse parents and subsequent selection over a number of generation to develop improved crop variety. This approach has several limitations such as requires long period (5–12 years) to develop crop variety, based on phenotypic selection (PS), high environmental noise, and less effective for complex and low heritable traits (Tuberosa, 2012). MAB involves the use of molecular markers for the indirect selection on trait of interest in crop species, requires minimum phenotypic information during training phase, and were initiated to solve limitations of conventional breeding (Collard and Mackill, 2008). The marker-assisted selection (MAS) and genomic selection (GS) are the two kinds of MAB. MAS use molecular markers known to be associated with trait of interest or phenotypes to select plants with desirable allele effecting target trait. It is efficient only for those traits that are controlled by fewer numbers of quantitative trait loci (QTLs) having the major effect on trait expression, whereas for complex quantitative traits which are governed by large number of minor QTLs, the method is even inferior to conventional phenotypic selection (PS; Zhao et al., 2014). The major reason is the estimation of QTL effects for minor QTLs through linkage mapping and genome-wide association mapping (GWAS) is often biased. Therefore, research communities were looking for solutions over decades how to deal with these complex traits and come out in the form of GS. GS estimates the genetic worth of the individual based on large set of marker information distributed across the whole genome, and is not based on few markers as in MAS. The GS develops the prediction model based on the genotypic and phenotypic data of training population (TP), which is used to derive genomic estimated breeding values (GEBVs) for all the individuals of breeding population (BP) from their genomic profile (Meuwissen et al., 2001) (**Figure 1**). The GEBVs allow us to predict individuals that will perform better and are suitable either as a parent in hybridization or for next generation advancement of the breeding program, because the molecular marker profile of those individual are similar to that of other plants of TP that have been recorded to perform better in the particular environments.

Since the concept of GS was proposed by Meuwissen et al. (2001) as an approach to predict complex traits in animals and plants, it is being only recently used in applied crop breeding. The most important reason behind this is the lack of cost-effective and high-throughput genotyping platforms, which is an essential requirement for GS. However, the next generation sequencing (NGS) has drastically reduced the cost and time of sequencing as well as single nucleotide polymorphism (SNP) discovery and has led to development of high-throughput genome-wide SNP genotyping platforms, especially the emergence of genotype-by-sequencing (GBS) which has resulted the implementation of SNPs suitable and affordable for GS in both model and non-model plant species (Poland et al., 2012). In this review, we try to make understand how NGS technologies will help to reap the true benefits of GS in the era of high-throughput genotyping for crop improvement.

## Genomic Vs. Phenotypic Selection

**FIGURE 2:**
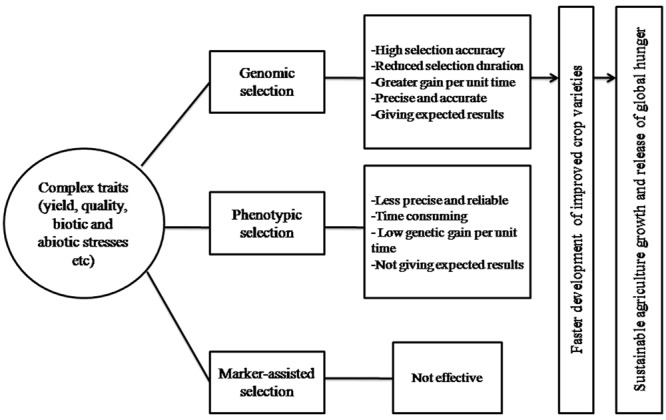
**Showing the different steps of GS for complex traits as well as its impact on agriculture growth and global hunger**.

The classical breeding has evolved dramatically in the last century and made significant contribution to crop improvement, developed high yielding and nutrient responsive semi-dwarf varieties of cereals during Green Revolution and hybrid rice in 1970s. These methods have produced the modern cultivars of almost all major crop species since the middle of 20th century and have achieved significant gains in terms of production and productivity. They pushed the yearly genetic gain of 1% increase in potential grain yield, which is not sufficient to keep pace with the world population growing at the rate of 2% per year, which relies heavily on crop products as source of food (Oury et al., 2012; Fischer et al., 2014). Moreover, the conventional breeding is based on PS to select better parents either for crossing or generation advancement and are less effective for low heritable multi-genic quantitative traits (yield, quality, biotic, and abiotic stresses), which are considerably influenced by environment and G × E interaction. In addition, these methods are challenged by being time consuming, laborious, require large land, cost ineffective, population size, and are less precise and reliable, hence necessitate the immediate, rapid and efficient selection systems for the development of high yielding and climate resilient crop varieties. Therefore, to address these challenges new strategies called GS based on reduced phenotyping, and were selection is based on marker/genotypic profile was suggested (Meuwissen et al., 2001). GS develops the prediction model by integrating the genotypic and phenotypic data of TP, which is subsequently used to calculate GEBV for all the individuals of BP from their genotypic data (Poland et al., 2012) (**Figure 1**). The GEBV is derived on the combination of useful loci that occur in the genome of each individual of the BP, and it provides a direct estimation of the likelihood of each individual to have a superior phenotype (i.e., high breeding value). Selections of new breeding parents are based on the estimated GEBV, which leads to shorter breeding cycle duration as it is no longer necessary to wait for late filial generations (i.e., usually F6 or following in the case of wheat) to phenotyping quantitative traits such as yield, biotic, and abiotic stresses etc. Given realistic assumptions of selection accuracies, breeding cycle times, and selection intensities, GS can increase the genetic gain per year compared to PS in both animal and crop breeding (Zhong et al., 2009; Heffner et al., 2010). Moreover, for those traits that have a long generation time or are difficult to evaluate (i.e., insect resistance, bread making quality, and others) GS becomes cheaper or easier than PS so that more candidates can be characterized for a given cost, thus enabling an increase in selection intensity. Hence, GS offers number of merits over PS by reducing selection duration, increasing selection accuracy, intensity, efficiency, and gains per unit of time, hence saves time, money and provides more reliable results as well as is environmentally insensitive (Rutkoski et al., 2011; Desta and Ortiz, 2014), thus enables the faster development of improved varieties of crop species to cope the challenges of climate change and decreasing arable land (**Figure 2**). One premise of using GS in applied breeding programs is the availability of high-density genome wide molecular markers at a cost that is comparable to (or lower than) the cost of phenotyping (Meuwissen et al., 2001; Goddard and Hayes, 2007; Heffner et al., 2009; Jannink et al., 2010).

## GS and Complex Traits

The inheritance of quantitative trait varied from simple to complex, simple quantitative traits inheritance are dominated by few major genes/QTLs whereas the complex traits are controlled by many minor effect genes distributed throughout the genome. Most of the economic traits in crop species are complex quantitative traits (e.g., yield, quality, biotic, and abiotic stresses etc), hence remain the main focus of plant breeders and researchers over the decades. These traits are constrained by their low heritability and environment sensitiveness; hence traditional breeding approaches were slow in targeting these traits that too under costly and labor intensive phenotyping (Bhat et al., 2015). MAS based on initial identification of marker-trait association either through linkage or Linkage Disequilibrium (LD) mapping was sometime thought to have potential for clearing genetic basis of complex traits when everywhere was slogans of QTL and QTL. But very soon it was recognized that MAS and association genetics were unable to capture the ‘minor’ gene effects that underpin most of the genetic variation in complex traits, and are inferior to phenotypic selection in case the associated marker account for small portion of genetic variation among the individuals of BP (Castro et al., 2003; Collins et al., 2003; Xu and Crouch, 2008) (**Figure 2**). The improvement of complex traits requiring multi-year and multi-location phenotypic evaluation to fix G ×E interaction is at times not feasible due to shortage of funds and labor. And what have been predicted over last two and half decades that molecular marker technology would reshape the breeding program and facilitate rapid gains from selection came finally true in the form of GS facilitated by cost-effective high-throughput NGS-based genotyping platforms. In contrast to traditional MAS approaches focusing on the identification and introgression of few major effect genes/QTLs, the GS considers all markers distributed throughout the genome to be incorporated into the model to generate a prediction that was the sum total of all genetic effects, regardless how many minor and major, and hence avoids missing of substantial portion of genetic variance contributed by loci of minor effects. The number of studies carried out earlier has shown GS models to be advantageous for complex quantitative traits viz., grain yield, quality, biotic and abiotic stresses etc (de los Campos et al., 2009; Crossa et al., 2010; Burgueño et al., 2012; González-Camacho et al., 2012; Jannink et al., 2010). The key feature of this approach is the genome-wide high density markers used potentially explain all the genetic variance, so that at least one marker is in LD with each QTL governing the trait of interest and the number of effects per QTL to be estimated is small. The most obvious advantage of GS is the genotypic data obtained from the seed or seedling can be used for predicting the phenotypic performance of mature individuals without the need for extensive phenotyping evaluation over years and environments, thus increasing the speed of varietal development in crop species. The approach is special thanks to especially NGS which make this approach feasible by discovering large number of SNPs and genotyping methods to genotype this huge SNP information across large BP. Hence, whole-genome prediction based selection will replace the phenotypic selection and marker assisted breeding protocols in the coming era for at least in complex traits that require least phenotyping for updating model to build up the prediction accuracy.

## Ngs: Key to the Success of GS

To sequence/re-sequence the entire genome (or part of it) of a large number of accessions is the ultimate approach for the study of polymorphism in any crop. This was not possible before the introduction of NGS platform, which has revolutionized genomics approaches to biology drastically increasing the speed at which DNA sequence can be acquired while reducing the costs by several orders of magnitude. NGS technologies have been widely used for whole genome sequencing (WGS), whole genome resequencing (WGRS), *de novo* sequencing, GBS, and transcriptome and epigenetic analysis (Varshney et al., 2009). However, there are few technical challenges to NGS technologies such as NGS data analysis is time consuming, requires sufficient knowledge of bioinformatics to harvest accurate information from these sequence data, short sequencing read lengths and data processing steps/bioinformatics (Daber et al., 2013). In the last few years, third generation sequencing (TGS) technologies were developed and are being used to improve NGS strategies. These technologies produce longer sequence reads in less time and that too at lower costs per instrument run. In the coming few years, TGS platform has been predicted to replace the SGS by 47% (Peterson et al., 2010). These technologies are also expected to increase the accuracy of SNP discovery, and reduce the chances of wrong base calling.

Initially, the WGS made available by Sanger sequencing was limited to few model plant species (rice, maize, *Arabidopsis* etc). The availability of WGS led considerable shift from fragment based polymorphism identification to sequence based polymorphism (SSR, SNP etc) identification to expedite the marker identification process and to increase the number of informative markers. The Sanger sequencing being time consuming, costly and provide information only to the target individual, has limited its use in specific gene discovery (Ray and Satya, 2014). Hence, it is not feasible for breeding programs involving large population size. The NGS technologies and powerful computational pipelines have driven the whole genome sequencing cost to drop by several folds allowing discovery, sequencing and genotyping of thousands of markers in a single step (Stapley et al., 2010). NGS has emerged as a powerful tool to detect numerous DNA sequence polymorphism based markers within a short timeframe, growing as a powerful tool for genomic-estimated breeding (GAB). Presently, the WGS of parental and progeny lines of mapping populations as well as of germplasm lines currently present in different germplasm repositories is costly and not feasible, but the moving revolution in NGS technologies can reduce the cost for resequencing the genome to only a few hundred US dollars. This will lead to the discovery of huge markers information to meet all the needs of plant breeding. By that time targeted sequencing seems to be more cost-effective option for large scale marker discovery, particularly in case of large and un-decoded genomes. Several targeted marker discovery techniques have been developed using NGS platforms viz., reduced-representation libraries (RRL), complexity reduction of polymorphic sequences (CRoPS), restriction-site associated DNA sequencing (RAD-seq), double digest RADSeq (ddRADSeq), ezRADSeq, 2bRADSeq, DArTSeq, genome reduction on restriction site conservation (GR-RSC), sequence based polymorphic marker technology, multiplexed shotgun genotyping (MSG), genotyping-by-sequencing (GBS), molecular inversion probe, solution hybrid selection and microarray-based GS, which involve partial representation of the genome and those can be utilized even in absence of prior knowledge on WGS (Toonen et al., 2013; Ray and Satya, 2014). Among these NGS technologies RAD-seq (or its variants) and GBS have already been proved to be effective for GAB and were frequently used in GWAS and GS studies (Yang et al., 2012; Glaubitz et al., 2014) (**Table 1**). The sequencing technology development closely follows Moore’s law (Wetterstrand, 2014), which indicates that WGS or NGS cost will drop by several magnitudes, and WGS will be preferred over targeted genome sequencing in near future (Marroni et al., 2012). We expect that overwhelming flow of WGS will not completely wipe off the partial genome sequencing approach, but it would be a preferred choice for short term projects for strengthening next generation plant breeding.

**Table 1:** Genomic selection (GS) efforts performed for various traits in different crops using different statistical models, software packages, and next-generation sequencing (NGS) marker genotyping platforms.

S.no.	Species	NGS marker platform	Trait	Population size	Total SNP markers	Prediction accuracy	Model	Software packages	Reference
1	Rice	GBS	Grain yield, flowering time	363	73,147	0.31–0.63	RR-BLUP	R package rrBLUP	Spindel et al., 2015
2	Rice	DArTseq	Grain yield, plant height	343	8,336	0.54	G-BLUP, RR-BLUP	BGLR and ASReml R packages	Grenier et al., 2015
3	Wheat	GBS	Stem rust resistance	365	4,040	0.61	G-BLUP B	R package GAPIT	Rutkoski et al., 2014
4	Wheat	GBS	Grain yield, plant height, heading date and pre-harvest sprouting	365	38,412	0.54	BLUP	R package rrBLUP	Heslot et al., 2013
5	Wheat	GBS	Grain yield	254	41,371	0.28–0.45	BLUP	ASReml 3.0	Poland et al., 2012
6	Wheat	GBS	Yield and yield related traits, protein content	1127	38,893	0.20–0.59	BLUP	rrBLUP version 4.2	Isidro et al., 2015
7	Wheat	GBS	Fusarium head blight resistance	273	19,992	0.4–0.90	RR-BLUP	R package GAPIT	Arruda et al., 2016
8	Wheat	GBS	Grain yield, protein content and protein yield	659	–	0.19–0.51	RR-BLUP	R package rrBLUP	Michel et al., 2016
9	Wheat	GBS	Grain yield	1477	81,999	0.50	G-BLUP	R package rrBLUP	Lado et al., 2016
10	Wheat	DArTseq	Grain yield	803	–	0.27–0.36	G-BLUP	BGLR and ASReml R packages	Pierre et al., 2016
11	Wheat	GBS	Grain yield, Fusarium head blight resistance, softness equivalence and flour yield	470	4858	0.35–0.62	BLUP	BGLR R-package	Hoffstetter et al., 2016
12	Wheat	GBS	Heat and drought stress	10819	40000	0.18–0.65	G-BLUP	BGLR R-package	Crossa et al., 2016
13	Maize	GBS	Drought stress	3273	58 731	0.40–0.50	G-BLUP	BGLR R-package	Zhang et al., 2015
14	Maize	GBS	Grain yield, anthesis date, anthesis-silkimg interval	504	158,281	0.51–0.59	PGBLUP, PRKHS	R Software	Crossa et al., 2013
15	Maize	GBS	Grain yield, anthesis date, anthesis-silkimg interval	296	235,265	0.62	PGBLUP, PRKHS	R software	Crossa et al., 2013
16	Maize	DArTseq	Ear rot disease resistance	238	23.154 Dart-seq markers	0.25–0.59	RR-BLUP	R package rrBLUP	dos Santos et al., 2016
17	Soybean	GBS	Yield and other agronomic traits	301	52,349	0.43–0.64	G-BLUP	MissForest R package, TASSEL 5.0	Jarquín et al., 2014b
18	Canola	DArTseq	Flowering time	182	18, 804	0.64	RR-BLUP	R package GAPIT	Raman et al., 2015
19	Alfalfa	GBS	Biomass yield	190	10,000	0.66	BLUP	R package, TAASEL software	Li et al., 2015
20	Alfalfa	GBS	Biomass yield	278	10,000	0.50	SVR	R package rrBLUP, R package BGLR, R package ‘RandomForest	Annicchiarico et al., 2015
21	Miscanthus	RADseq	Phenology, biomass, cell wall composition traits	138	20,000	0.57	BLUP	R package rrBLUP	Slavov et al., 2014
22	Switchgrass	GBS	Biomass yield	540	16,669	0.52	BLUP	glmnet R package, R package rrBLUP	Lipka et al., 2014
23	Grapevine	GBS	Yield and related traits	800	90,000	0.50	RR-BLUP	R package BLR, R package rrBLUP	Fodor et al., 2014
24	Intermediate wheatgrass	GBS	Yield and other agronomic traits	1126	3883	0.67	RR-BLUP	R package rrBLUP, BGLR R-package	Zhang et al., 2016
25	Perennial ryegrass	GBS	Plant herbage dry weight and days-to-heading	211	10,885	0.16–0.56	RR-BLUP	R software	Faville et al., 2016

**FIGURE 3:**
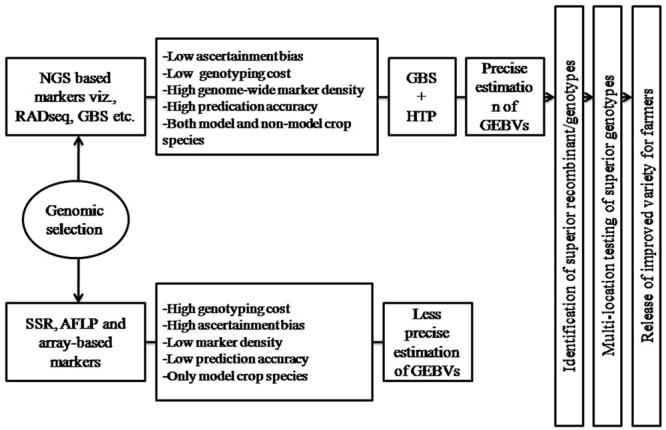
**Role of next-generation sequencing (NGS) based marker technologies and high-throughput phenotyping (HTP) on GS**. Both NGS and HTP occupy a critical position in the precise estimation of GEBV that predict the breeding value of individuals in a breeding population using GS.

The NGS-based marker technologies provide genome-wide marker coverage at a very low cost per data point and have increased the speed, throughput, and cost effectiveness of genome-wide genotyping, thus allowing us to assess the inheritance of the entire genome with nucleotide-level precision. Previously, to generate marker data were expensive and laborious, and number of markers were major constrain for MAB strategies that could efficiently be assayed. This restricted the use of markers only in critical genomic regions to predict the presence or absence of agriculturally important traits. But, the expansion of NGS technologies and genotyping platforms widen the marker applications for crop improvement and were the basis for the success of GS, which has almost shifted the complete reliance on phenotyping to an increased reliance on genotyping-based selection. The NGS-based genotyping offer number of benefits over array-based genotyping such as low genotyping cost (per sample cost <$20 USD), low ascertainment bias, increased dynamic range detection offered by sequencing in polyploid species, insight into non-model genomes were no a priori genomic information is available and high marker density, hence made them the method of choice in genotyping for GS (Poland et al., 2012) (**Figure 3**). Among the number of factors that ascertain the efficiency and accuracy with which the superior lines can be predicted through GS, the type and density of marker used as well as size of reference population (limited by high cost genotyping) are the most critical factors (Jannink et al., 2010; Lorenz et al., 2011), both have been resolved by NGS genotyping technologies (Jarquín et al., 2014a). In addition, the population structure (i.e., genetic relatedness) is another key factor affecting predictions of breeding values with genomic models and could result in biased accuracies of genomic predictions (Saatchi et al., 2011; Riedelsheimer et al., 2013; Wray et al., 2013). Population structure produces spurious marker-trait associations in genome-wide association studies due to different allele frequencies among subpopulations, which may inflate estimate of genomic heritability and bias accuracies of genomic predictions (Price et al., 2010; Visscher et al., 2012; Wray et al., 2013). When population structure exists in both training and validation sets, correcting for population structure led to a significant decrease in accuracy with genomic prediction. In comparison to SSR and array based marker systems, the NGS-based marker genotyping provide abundant SNP information across whole crop genome to accurately estimate the population structure of TP, which in turn is used to train the model that accurately predict the GEBV of BP (Isidro et al., 2015).

Thus, the rapid advances in sequencing technology have led to higher throughput and low cost per sample, and positioning NGS-based genotyping as a cost-effective and efficient agrigenomics tool for performing GS in both model and non-model crop species as well as for crops with large and complex genomes (Metzker, 2010; Kirst et al., 2011; Poland et al., 2012; Toonen et al., 2013) (**Table 1**). The NGS genotyping have been reported to increase GEBV prediction accuracies by 0.1 to 0.2 over other established marker platform in cereals and other crop species (Poland et al., 2012). GS have been attempted in *Miscanthus sinensis* for 17 traits related to phenology, biomass, and cell wall composition using RADSeq, and genome-wide prediction accuracies were investigated to be moderate to high (average of 0.57) and suggested immediate implementation of GS in *Miscanthus* breeding programs (Slavov et al., 2014). Spindel et al. (2015) also revealed the prediction accuracies of 0.63 for flowering time, by studying the GS in tropical rice breeding lines. In addition, the NGS marker genotyping have been reported to give higher GS accuracy than DArT markers on the same lines of wheat (*Triticum aestivum* L.), despite 43.9% missing data (Heslot et al., 2013). These entire make the sequence based genotyping an ideal approach for GS and its successful application in crop breeding (**Figure 3**).

## GS and GBS

Genotype-by-sequencing follows a modified RAD-seq based library preparation protocol for NGS and is a simple and highly multiplexed system. The important feature of this system include reduced sample handling, fewer PCR and purification steps, low cost, no reference sequence limits, no size fractionation and efficient barcoding technique (Davey et al., 2011). The recent advances in NGS have reduced the DNA sequencing cost to the point that GBS is now feasible for large genome species and high diversity (Elshire et al., 2011). It enables the detection of thousands of millions of SNPs in the large collections of lines that can be used for genetic diversity analysis, linkage mapping, GWAS, GS, and evolutionary studies. (Beissinger et al., 2013). GBS is becoming increasingly important as a cost-effective and unique tool for genomics-assisted breeding in a range of plant species. Genotyping-by sequencing combines marker discovery and genotyping of large populations, making it an excellent marker platform for breeding applications even in the absence of a reference genome sequence or previous polymorphism discovery. The GBS method offers a greatly simplified library production procedure more amenable to use on large numbers of individuals/lines (Elshire et al., 2011). The original GBS protocol utilizing only one enzyme ApeKI have been modified in plants by two-enzyme (PstI/MspI) GBS protocol, which allows greater reduction of complexity and uniform library for sequencing, and have been applied in wheat and barley (Poland et al., 2012). In crop species with large and complex genomes as well as lack of reference sequence the marker technologies lagged behind, which is an important factor to consider for large scale application of GS in crop plants. The high polyploidy level, large genome size and lack of reference genome (wheat) were the major hindrance of molecular marker development in the crop species. Genotyping-by-sequencing has recently been applied to large complex genomes of barley (*Hordeum vulgare* L.) and wheat (*Triticum aestivum* L.), and shown to be an effective tool to rapidly generate molecular markers for these species (Poland et al., 2012). The GBS have also been used for de novo genotyping of breeding panels and to develop accurate GS models, for the large, complex, and polyploid wheat genome. GAB value prediction accuracies were 0.28 to 0.45 for grain yield, an improvement of 0.1 to 0.2 over an established marker platform for wheat (Heslot et al., 2013). The first evidence of the prediction accuracy of GBS in plants came from Poland et al. (2012), who showed good accuracy using GBS in prediction models for polyploid wheat breeding, and from Crossa et al. (2013), who predicted doubled-haploid maize lines using pedigree as well as imputed and unimputed GBS data. In these applications, read depth as low as ∼1x was sufficient to obtain accurate EBV without using imputation and error correction methods. Since then GS involving GBS have been reported in multiples of crop species including both model and non-model (**Table 1**). In soybean, prediction accuracy for grain yield, assessed using cross validation, was estimated to be 0.64, indicating good potential for using GS for grain yield in soybean (Jarquín et al., 2014b). The GBS has the potential to drive the cost per sample below $10 through intensive multiplexing. Genotyping cost of GBS per individual is lowest in comparison to array-based and other NGS-based markers in wheat and other non-model crop species (Bassi et al., 2016). The fraction of the genome covered by GBS can potentially be much greater than the fraction captured by even the densest SNP arrays currently available in crop plants (Gorjanc et al., 2015). Furthermore, unlike SNP arrays that are typically developed from a limited sample of individuals, GBS can capture genetic variation that is specific to a population or family of interest. GBS has the advantage that markers are discovered using the population to be genotyped, thus minimizing ascertainment bias. Hence, the flexibility, low cost and GEBV prediction accuracy of GBS make this an ideal approach for GS (**Table 1**; **Figure 3**).

## GS: Implications in Crop Improvement

The applied plant breeding is the ultimate source of improved crop varieties, and has led to green revolution in 1960s. At every time this field was supported and facilitated by the new technologies and approaches. The impact of climate change on crop production and global food security is being discussed currently throughout the world (Reynolds, 2010). The population of the world is expected to rise by 50% till 2050 (Tester and Langridge, 2010), requiring 70% increase in crop production (Furbank and Tester, 2011). Therefore, to fight against these challenges and maintaining sustainable agriculture, new crop varieties are required at an accelerated rate to increase production as well as withstand better biotic and abiotic stresses. As discussed that most of the agriculturally important traits are governed by minor effect genes, and with a high occurrence of epistatic interactions such as grain yield, plant growth and stress adaptation etc (Mackay, 2001). Improvement of these traits through conventional breeding and MAS do not met the expected results to pace with growing human population. In this regard, GS provides new opportunities for increasing the efficiency of plant breeding programs (Bernardo and Yu, 2007; Heffner et al., 2009; Crossa et al., 2010; Lorenz et al., 2011). The GS has the potential to fix all the genetic variation and has ability to accurately select individuals of higher breeding value without the requirement of collecting phenotypes pertaining to these individuals. This has facilitated a shortening of the breeding cycle and enable rapid selection and intercrossing of early generation breeding material (**Figure 2**). Recent research has shown that GS has the potential to reshape crop breeding, and many authors have concluded that the estimated genetic gain per year applying GS is several times that of conventional breeding (Bassi et al., 2016). The cost of genotyping has declined dramatically in the era of NGS (Davey et al., 2011), whereas the cost of phenotyping is increasing due to labor and land-use expenses, and has led to increased utility of GS in crop improvement. This will expand the genetic evaluation of germplasm in crop improvement programs and accelerate the delivery of crop varieties with improved yield, quality, biotic and abiotic stress tolerance, and thus directly benefit attempts to address the challenge of increasing global hunger. Thus, GS will be the cornerstone for the release of global hunger, and has tremendous impact on crop breeding and variety development (**Figure 2**).

## GS and High Throughput Phenotyping (HTP)

It is clear from the above discussion that genotyping no more limit the prediction accuracy of GS. But the technical challenge in implementing the GS in crop plants is the reliability of phenotypic data that creates genotype-phenotype gap (GP gap). The GS predication model used to derive GEBV for all genotyped individuals of the reference set depends upon the precision and accuracy the phenotypic data is taken on TP, and thereby the genetic gain achieved after every generation of selection (Meuwissen et al., 2001). The precise phenotypic data is one of the key components to train GS model for accurately predicting GEBV of BP (Cabrera-Bosquet et al., 2012). In this regard, several phenotyping facilities have been developed around the world that can scan and record precise and accurate data for thousands of plants quickly by making use of non-invasive imaging, spectroscopy, image analysis, robotics and high-performance computing facilities (Cobb et al., 2013). The HTP helps us to collect high quality accurate phenotyping data. The manual, invasive and destructive methods of plant phenotyping were laborious, costly and less precise, often led inaccuracy in GAB as well as limit the population size. This importance can be realized by the fact that an International Plant Phenomics Initiative was launched recently to address crop productivity^1^. The earlier manual methods of plant phenotyping are now giving way to high-throughput precise non-destructive imaging techniques. These phenomics facilities make sure to scan thousands of plants in a day so that this phenotyping technology will become similar to high-throughput DNA sequencing in the field of genomics (Finkel, 2009). Hence, to achieve fruitful results from GS and GAB much more efforts and funds are required to be allocated in this field. In India well established phenomic facility has been not created yet, therefore efforts are required to create such facility in the country to boost agriculture production. Hence, HTP will change the paradigm of GS and led its effective application in crop plants as well as harness its true benefits for crop improvement (**Figure 3**).

## Conclusion

The classical breeding had made a significant contribution to crop improvement but was slow in targeting the complex and low heritable quantitative traits. In this regard, GS has been suggested to have a potential to fix all the genetic variation of complex traits. Many studies have shown tremendous opportunities of GS to increase genetic gain in plant breeding. The important consideration for GS to work in crop plant is the availability of low cost, flexible and high density marker system. Revolution of inexpensive NGS technologies has resulted in increasing number of crop genomes as well as provides the low cost and high density SNP genotyping. These marker technologies have deeply estimated the population structure of both training and validation set, and have increased the selection accuracy of GS. The NGS markers, as well as methodological refinements (such as the implementation of genotype-by-environment interaction in prediction models), are notably contributing to paving the way for a successful implementation of GS in plant breeding. Hence, GS will be the key approach for the success of second “Green Revolution” to occur. Furthermore, the GS and HTP together will change the entire paradigm of plant breeding as well as led to the effective increase in genetic gain for complex traits. In the future when the genomic sequencing cost further decreases and WGS become feasible and cost effective for GS, there will be further increase in the prediction accuracy of GS. Till that time matures the targeted sequencing seems to be more cost-effective option for large scale marker discovery and GS, particularly in case of large and un-decoded genomes.

## Author Contributions

JB, RS, NJ, PS, GS: conceived and designed the experiment. JB, SA, ZM, AT, MM, NJ, SD, VJ: collected the literature for this review. JB, SA, ZM, AT, MM, NJ, SD, VJ: wrote the manuscript draft. RS, PS, GS: edited this MS. All authors viz., JB, SA, ZM, AT, RS, MM, NJ, PS, GS, SD, VJ, KP: give final shape to this manuscript.

## Conflict of Interest Statement

The authors declare that the research was conducted in the absence of any commercial or financial relationships that could be construed as a potential conflict of interest.

## Footnotes

1 http://www.plantphenomics.org/
